# Hepatic and renal toxicity following the injection of copper oxide nanoparticles (CuO NPs) in mature male Westar rats: histochemical and caspase 3 immunohistochemical reactivities

**DOI:** 10.1007/s11356-022-21521-2

**Published:** 2022-06-23

**Authors:** Wael A. M. Ghonimi, Mosaid A. Z. Alferah, Naief Dahran, Eman S. El-Shetry

**Affiliations:** 1 Department of Histology and Cytology, Faculty of Veterinary Medicine, Zagazig University, Zagazig, 44519 Egypt; 2 Department of Biology, College of Science and Arts, Unaizah, Qassim University, Buraydah, Saudi Arabia; 3 Department of Anatomy, Faculty of Medicine, University of Jeddah, Jeddah, Saudi Arabia; 4 Department of Human Anatomy and Embryology, Faculty of Medicine, Zagazig University, Zagazig, Egypt

**Keywords:** Nanoparticles, Renal toxicity, Hepatotoxicity, Steatosis, Necrosis, CuO NPs

## Abstract

Copper nanoparticles are widely utilized in a variety of applications, including metal catalysts, semiconductors, heat transfer fluids in machine tools, and even in antibacterial medications. Forty mature healthy Westar rats were utilized in the current investigation and grouped randomly into four groups (*n* = 10 rats/group). Group I (G1) was kept as a control group, but G2, G3, and G4 were intraperitoneally injected with CuO NPs with a dose (5 mg, 10 mg, 25 mg/kg body weight/day) respectively for 9 days. Rats were sacrificed; then, the livers and kidneys were dissected and subjected to histopathological and immunohistochemical examination. Our findings of G2 and G3 revealed mild to moderate degenerative changes within the hepatic parenchyma, moderate blood vessel congestions, glycogen depletion, hemosiderosis, and microvesicular steatosis (fatty changes within the hepatocytes). In addition, at the level of kidney, our examination clarified moderate degenerations of the renal corpuscles and renal tubules with moderate swelling and congestions of the glomerulus with moderate vacuolations in the renal tubules lining epithelium. On the other hand, increasing the dose of CuO NPs, the toxicity became more obvious, where the liver of G4 revealed severe necrosis of hepatocytes with completely disorganizations of the hepatic rays, loss of the hepatic architectures, severe steatosis, severe hemosiderosis, sinusoidal dilatations with congestions, as well as severe fibrous tissue proliferation with anti-inflammatory cell infiltrations specially around portal triad with hyperplasia of bile duct. Meanwhile in kidney, G4 clarified severe necrosis and atrophy of the renal corpuscles with severe damage of Bowman’s capsule leading to completely disorganization and loss of normal renal cortex architectures, severe congestion of the glomerulus, severe necrosis of the renal tubules with damage and sloughing for its lining epithelium, and severe hemorrhage between renal tubules. In addition, severe and diffuse caspase 3 immunoreactivity were observed within the hepatic and renal tissues of G4. The present investigation was concluded that the CuO NPs have a potential toxicological effect on the hepatic and renal tissues that may affect their functions.-->

## Introduction

Nanotechnology is an emerging field of science which involves synthesis and development of several nanomaterials (Dubchak et al. 2010).

Nanoparticles (NPs) can be recognized as objects or materials that ranging in size from 1 to 100 nm (ISO/TS 2008; Dubchak et al. 2010).

They have very specific physical and chemical properties of shape, size, and high ratio of surface area to volume. So, NPs were involved in several applications for numerous biological and medical cases (Erb et al. 2002; Roduner 2006).

Because of their small size, NPs have the ability to enter and penetrate physiological barriers. And also, they travel via the circulatory systems to all body organs affecting the human and animal’s health (Wang et al. 2007; Shirvani et al. 2014).

Metal oxide nanoparticles (NPs) belong to a family of nanomaterials and are generated using copper, gold, silver, zinc, magnesium, alginate, and titanium. Nanoparticles are applied for several purposes like medical treatments, also in different industry productions as oxide and solar fuel batteries for the energy storage, to broad combination into different materials for daily utilization such as clothes or cosmetics (Dubchak et al. 2010; Chang et al. 2012). Furthermore, metal oxide NP retention in the environment and food chain is high and the continuous exposure to them may affect human health (De Berardis et al. 2010).

Recently, metal oxide nanoparticles have been receiving a considerable attention because of their potential uses in nanosensors, optoelectronics, nanoelectronics, nanodevices, catalysis, and information’s storage. CuO among various metal oxide NPs has gained a particular attention because it is the simplest member of the copper compound family and exhibits a variety of useful physical properties including electron correlation effects, high temperature superconductivity, and spin dynamics (El-Trass et al. 2012).

CuO NPs are also used in a variety of technological applications, including electronics, wood protection, catalysts, and antimicrobial products (Ben-Moshe et al. 2009; Ren et al. 2009; Ahamed et al. 2014; Bhaumik et al. 2014). They are also used as coatings in food packaging (Longano et al. 2012) and ink additives (Zenou et al. 2014). And also, because of premium mending effects of the copper nanoparticles, they are added as an additive into the lubricant oil to minimize wear and friction effectively, or to repair a worn surface (Liu et al. 2004).

Moreover, copper oxide NPs have strongly superconductive properties, and so they are involved in various consumer electronics including batteries, solar cells, heat transfer fluids, catalysis, and gas sensors (Filipic and Cvelbar 2012; Bondarenko et al. 2013). Cu NPs are also utilized as one of the principle constituents of herbicides, algaecide, and fungicides; however, they have the ability to cause oxidative DNA damage and genotoxicity at the cellular level (Song et al. 2012), where they can interact with subcellular organelles after crossing the plasma membrane, causing oxidative stress (Fahmy and Cormier 2009; Wang et al. 2011; Melegari et al. 2013).

Some researchers clarified that CuO NPs were extremely hazardous and toxic when compared with other metal oxide nanoparticles and they can accumulate in the body tissues (Karlsson et al. 2008; Heinlaan et al. 2008).

Recently, the utilization of the NPs in human activities is elevated and widely distributed. So, clarifying the biological effects of different NPs and nanomaterial on animal and human organs is requiring distinct attentiveness. The main concern is the NP toxicity and the prospective hazard of NPs and their related products on the human health. CuO NPs are widely utilized in a variety of applications, including metal catalysts, semiconductors, heat transfer fluids in machine tools, and even in antibacterial medications (Wang et al. 2004; Aruoja et al. 2009).

The aim of our work is to clarify and evaluate the possible histological changes as a result of the toxicological effects of the copper oxide nanoparticles (CuO NPs) on Westar rat’s liver and kidney.

## Materials and methods

### Animals and housing

The current investigation had been conducted on forty apparently healthy mature male Westar rats that obtained from the Laboratory Animal Unit, Faculty of Pharmacy, Unaizah, Qassim University, Saudi Arabia. Animal’s weights were 150 ± 20 g and with average 3 months age. Rats were housed in a controlled environment with ideal conditions such as a constant temperature of 20 to 23 °C and a light-dark cycle (14 h of light and 10 h of dark was fixed throughout the experiment). Under hygienic conditions, the rats were kept in transparent polypropylene cages, 10 rats/cage, with free access to water and dry rat pellet feeds. The rats were allowed to acclimatize for a week before starting the experiment for accommodation.

### Supplements (nanoparticles)

Well-scattered copper oxide nanoparticles (nanopowder with an average size <50 nm) at 50 wt% in distilled water (Sigma, Aldrich) were utilized in the current investigation. NP dispersion was characterized with pH 5.5 ± 0.1, concentration 50 wt% in H2O, and density 1.7 g/ml ± 0.1 g/ml. And also, they had crystalline shape with length <50 nm, diameter <50 nm, and surface area 29 m^2^/g.

### Experimental protocol

Forty mature male Westar rats were grouped randomly into four groups (*n* = 10 rats/group). They were subjected for 9 days to one of the following treatments:

- Group I (G1): rats were kept as a control and fed with a basal diet without CuO NP injection for 9 days.
- Group II (G2): rats were injected CuO NPs (5 mg/kg body weight/day; intraperitoneally) for 9 days.
- Group III (G3): rats were injected CuO NPs (10 mg/kg body weight/day; intraperitoneally) for 9 days.
- Group IV (G4): rats were injected CuO NPs (25 mg/kg body weight/day; intraperitoneally) for 9 days.

### Histological and histochemical processing

At the end of experiment, cervical dislocation of rats and for histological analysis, the liver and kidney were separated immediately and small pieces from them were taken and fixed in Bouin’s solution firstly then transferred to neutral buffered formalin 10% for 48 h. The specimens were then dehydrated in series of ascending grades of ethanol, after that were cleared by xylene and infiltrated in soft melted paraffin in a hot air oven, and embedded in hard paraffin wax forming paraffin blocks. The paraffin blocks were transversely sectioning to the desired thickness of 4–5 μm by using a rotatory microtome and the sections were mounted on a glass slides. The obtained sections were stained with Harris’s hematoxylin and eosin (H&E) for routine histological studies, mercuric bromophenol blue for protein evaluation, Perls Prussian blue reaction to detect an excess of iron deposits such as hemosiderin deposits (hemosiderosis) in liver tissue, and Sudan black for the staining of a wide variety of lipids such as phospholipids, sterols, and neutral triglycerides; confirming fatty changes, blue Masson’s trichrome for demonstration of collagen fibers and cell cytoplasm, periodic acid–Schiff (PAS) for detection of glycogen and neutral muco-polysaccharides. All these histological and histochemical stains were according to Bancroft and Gamble (2008).

### Caspase 3 immunohistochemistry

Immunohistochemical staining was performed on 5-μm, formalin-fixed, paraffin-embedded sections using caspase 3 antibodies as a marker of programmed cell death (apoptosis) through the streptavidin-biotin technique. Deparaffinized sections were stained by an indirect immunoperoxidase technique (Polak and Noorden 1997).

Antigen retrieval was carried out through immersing the sections with 0.1 M citrate buffer solution (pH = 6) for 10 min using a microwave (600 W). For eliminating the activity of endogenous peroxidase, sections were incubated with 3% hydrogen peroxide (H2O2) in absolute methanol for 30 min at 4 °C. For minimizing nonspecific labeling, sections were incubated with blocking solution that was formed from phosphate buffer saline (PBS) containing 10% normal goat serum (NGS; Life Technology, Germany) and 0.1% Triton-X-100 (Sigma, Germany). The sections were incubated at 4 °C overnight with the specific primary antibodies diluted in blocking using caspase 3 antibodies at 1:1000 dilution (Sigma, Germany). Sections were incubated with biotin-conjugated secondary antibody at room temperature for 1 h in PBS containing biotinylated goat anti-rabbit IgG (1:300; Molecular Probes). After that, sections were washed in PBS 3 times × 5 min for each and subsequently incubated with streptavidin-peroxidase for 30 min at approximately 25 °C.

The final chromogen (streptavidin-biotin complex; immunopositive reactions) were developed and visualized using a 3,3′-diaminobenzidine tetrahydrochloride (DAB)-H2O2 solution, pH 7.0, for 3 min according to the package insert, which produces an insoluble brown pigment. The sections were then counter stained with Harris hematoxylin and dehydrated with ascending grades of ethanol, cleared with Rothihistol and mounted using Entellan (Merck, Germany).

The stained sections were photographed by a digital camera (Canon) connected to a light microscope (Zeiss) in the Histology and Cytology Department, Zagazig University, Zagazig, Egypt.

## Results

**Fig. 1:**
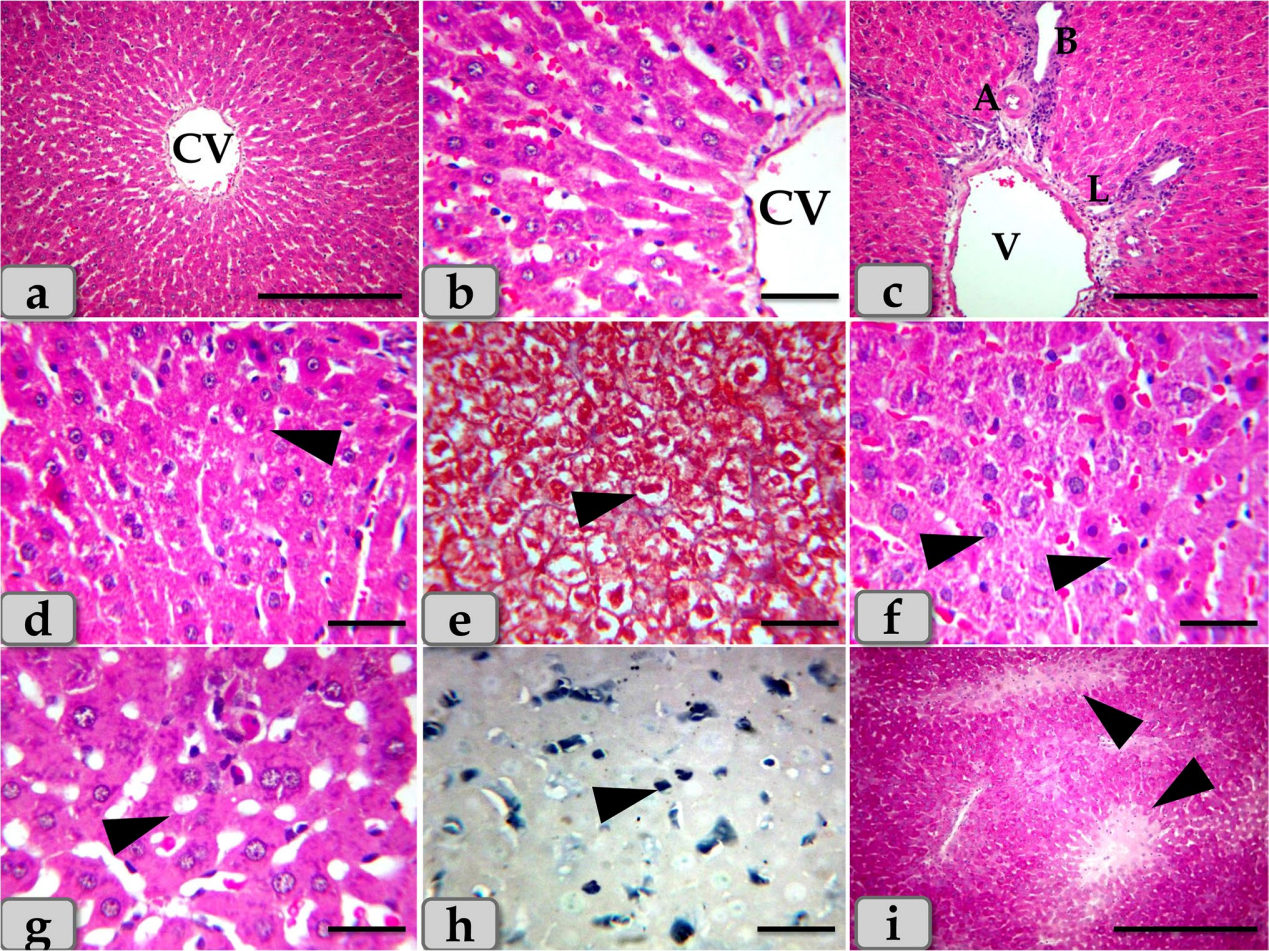
**a**, **b**, **c** Photomicrographs of the mature Westar rats’ liver of the control group (G1), **a** showing normal, intact hepatic parenchyma; classic hepatic lobules that bounded centrally with central vein (CV). **b** Showing normal, intact hepatocytes with irregular polygonal shaped and with single central spherical nucleus, intact hepatic rays or cords that dorsally radiated from the central vein to the lobule periphery, intact sinusoids in between hepatocytes. **c** Showing normal, intact portal triad that housing intact branches of hepatic artery (A), portal vein (V), bile ductules (B), and lymph vessel (L). **d**–**i** Photomicrographs of G2 and G3 that injected intraperitoneal with CuO NPs in a dose of 5 mg/kg bwt and 10 mg/kg bwt for 9 days, respectively. **d** and **e** Showing mild to moderate necrosis of the hepatic parenchyma with disorganization of the hepatic rays (arrow head). **f** Showing pleomorphic nuclei with various size and activity within the hepatocytes (arrow head). **g** Showing vacuoles with various shape and size distributing within the hepatocyte cytoplasm (arrow head). **h** Showing moderate fatty changes; steatosis within the hepatocyte cytoplasm (arrow head). **i** Showing pale patches of glycogen depletion within the parenchyma (arrow head). Stain: **a**, **b**, **c**, **d**, **f**, **g** H&E, **e** blue Masson’s trichrome, **h** Sudan black, **i** PAS. Scale bars: **a**, **c**, **i** = 300 μm and **b**, **d**, **e**, **f**, **g**, **h** = 40 μm

The histological examination of the mature Westar rats’ liver of the control group (G1) clarified normal, intact, homogenous hepatic parenchyma without any pathological changes. The hepatic parenchyma was composed mainly from numerous classic hepatic lobules. These lobules were hexagonal shaped and separated from each other by very thin connective tissue septa that making the demarcation between the lobules very difficult, so the hepatic lobules were not clear and all the hepatic parenchyma were appeared consisting of one lobe (Fig. 1a). These hepatic lobules were centrally bounded with the central vein (Fig. 1a, b) and peripherally with portal area or triad that housing intact branches of hepatic artery, portal vein, bile duct, nerve, and lymph vessel (Fig. 1c). The main components of each lobule were the hepatocytes that appeared irregular polygonal in shape with single, central, spherical, vesicular nucleus, however, some of them observed bi-nucleated. These hepatocytes were regulated in the form of hepatic rays or cords that dorsally radiated from the central vein to the lobule periphery. In addition, intact hepatic sinusoids with intact lining epithelium were also appeared circulating among the hepatic rays supplying the hepatocytes (Fig. 1b).

**Fig. 2:**
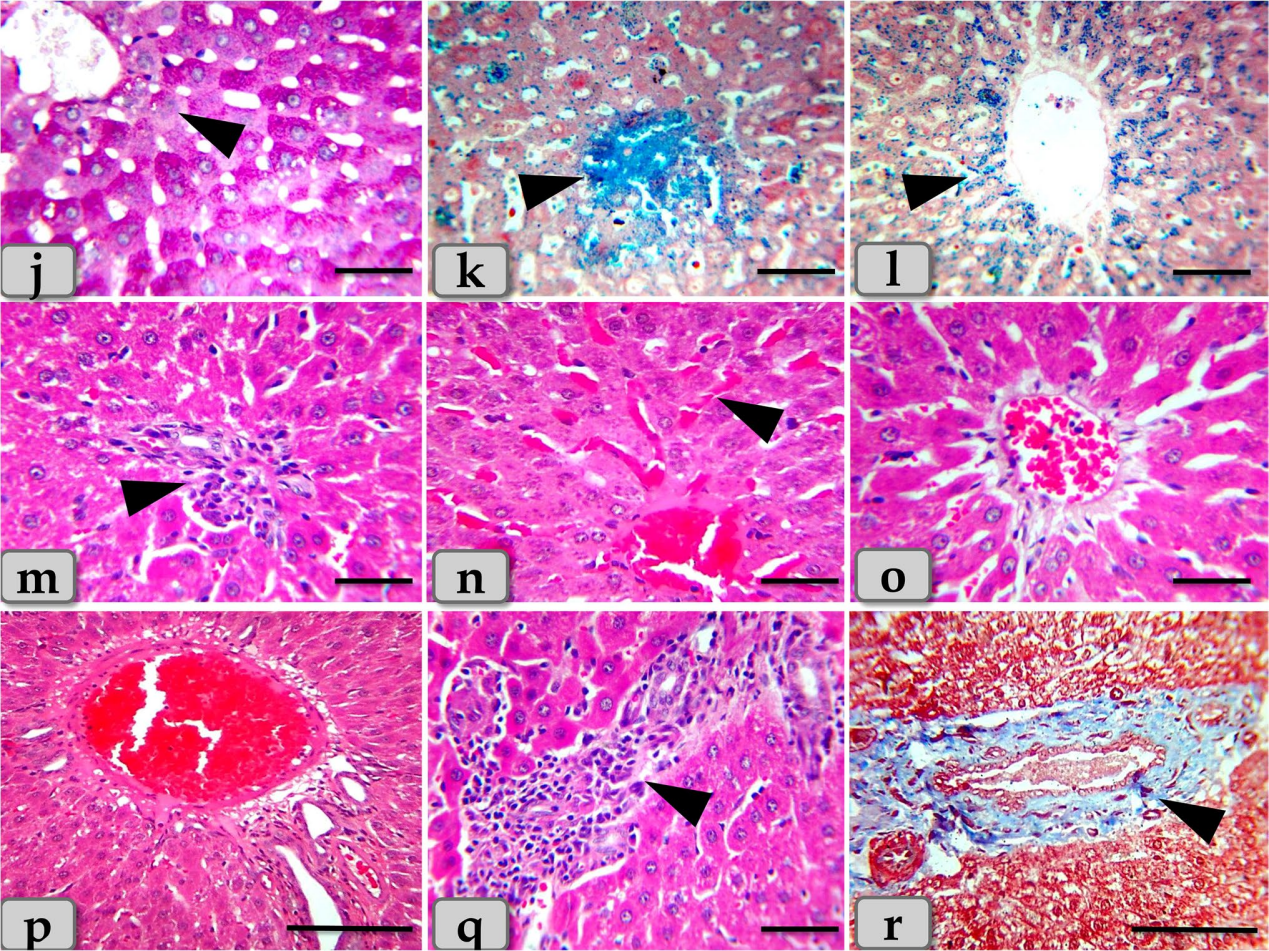
**j**–**r** Photomicrographs of G2 and G3, **j** showing moderate glycogen depletion within the hepatocyte cytoplasm (arrow head). **k** Showing patches of hemosiderosis (hemosiderin pigment precipitation) distributing within the hepatic parenchyma (arrow head). **l** Showing moderate hemosiderin deposits within the hepatocyte cytoplasm (arrow head). **m** Showing moderate chronic inflammatory cell infiltrations within the hepatic parenchyma (arrow head). **n** Showing moderate sinusoidal dilatation with congestion (arrow head). **o** Showing moderate congestion of central vein with moderate necrosis of its lining epithelium. **p** Showing moderate to severe dilatation and congestion of portal vein within the portal triad. **q** Showing moderate chronic inflammatory cell infiltrations within the portal triad (arrow head). **r** Showing moderate hyperplasia of bile duct with moderate necrosis of its lining epithelium and surrounding with chronic inflammatory cell infiltrations and fibrous connective tissue proliferations; moderate fibrosis (arrow head). Stain: **j** PAS, **k**, **l** Perls Prussian blue, **m**–**q** H&E, **r** blue Masson’s trichrome. Scale bars: **j**, **k**, **l**, **m**, **n**, **o**, **q** = 40 μm and **p**, **r** = 200 μm

Meanwhile, the histological examination of the liver of G2 and G3 injected intraperitoneal with CuO NPs in a dose of 5 mg/kg bwt and 10 mg/kg bwt for 9 days respectively characterized with mild to moderate necrosis of the hepatic parenchyma with disorganization of the hepatic rays (Fig. 1d). Furthermore, the necrotic hepatocytes were distinguished with enlarged cell, pyknotic nucleus, and pale cytoplasm (Fig. 1e). However, pleomorphic nuclei with various size and activity: some euchromatic and other heterochromatic within the hepatocytes were also observed (Fig. 1f). And also, vacuoles with various shape and size were observed distributing within the hepatocyte cytoplasm (Fig. 1g). The vacuole contents were reacted positively with Sudan black stain confirming fatty changes; microvesicular steatosis; fat droplet aggregations within the hepatocyte cytoplasm (Fig. 1h). With PAS stain, pale patches of glycogen depletion within the parenchyma were also identified (Figs. 1i and 2j). With Perls Prussian blue stain, moderate hemosiderosis (hemosiderin pigment precipitation) within the hepatocyte cytoplasm was clarified (Fig. 2k, l). Sometime, patches of hemosiderosis distributing within the hepatic parenchyma were appeared (Fig. 2k). Furthermore, focal inflammatory cells were observed infiltrated within the hepatic parenchyma (Fig. 2m). In addition, moderate sinusoidal dilatation with congestion (Fig. 2n) and moderate congestion of central vein with moderate necrosis of its lining epithelium (Fig. 2o) were also demonstrated.

Regarding the portal triad, some examined sections clarified moderate to severe dilatation and congestion of the portal vein (Fig. 2p), moderate chronic inflammatory cell infiltrations (Fig. 2q), and moderate hyperplasia of bile duct with moderate necrosis of its lining epithelium, surrounding with chronic inflammatory cell infiltrations with fibrous connective tissue proliferations; moderate fibrosis (Fig. 2r).

**Fig. 3:**
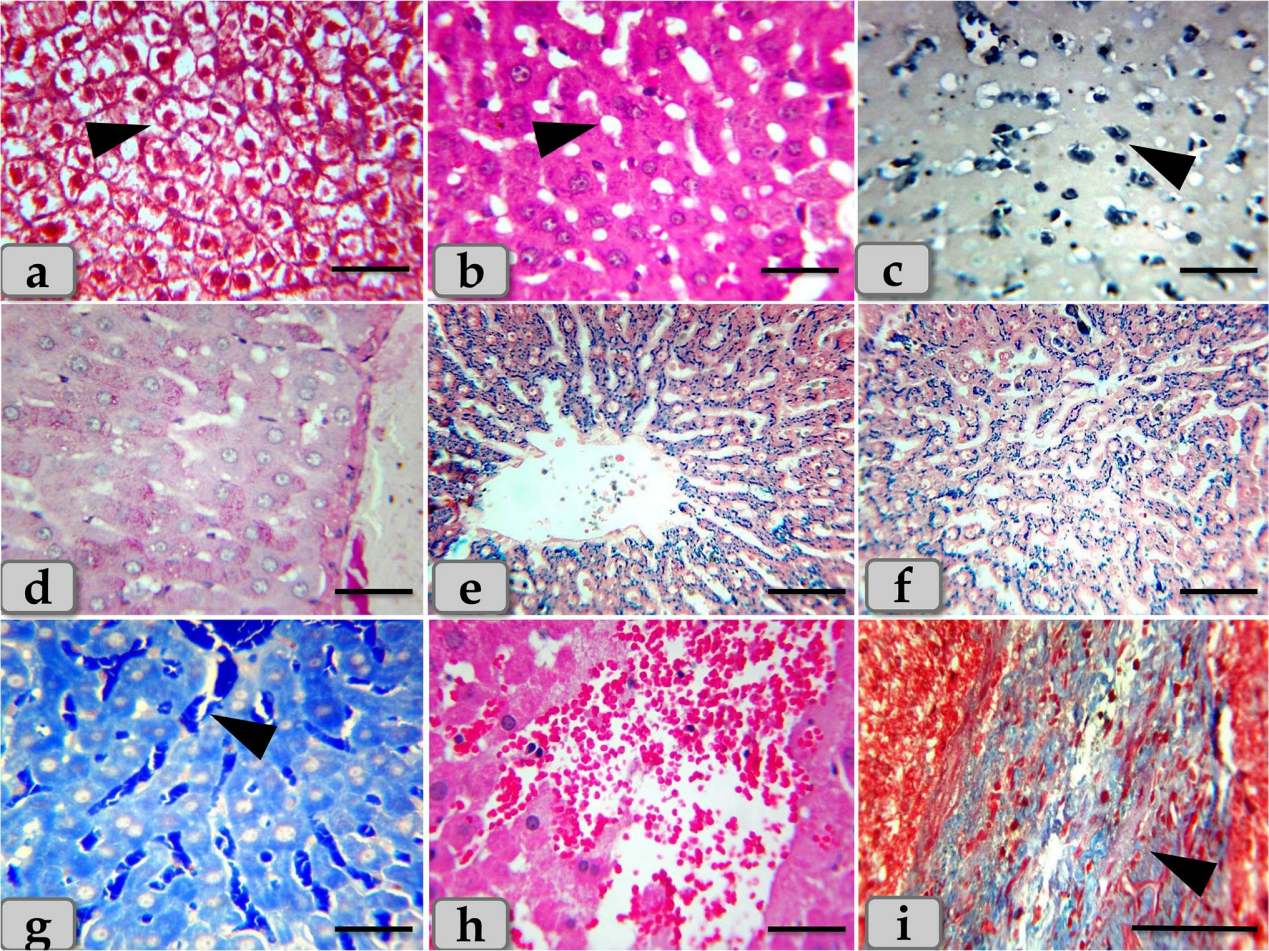
Photomicrographs of the G4 that injected intraperitoneal with CuO NPs in a dose of 25 mg/kg bwt for 9 days. **a** Showing severe necrosis of the hepatocytes with completely disorganization of the hepatic cords (arrow head). **b** Showing severe vacuolations of various shape and size distributing within the hepatocyte cytoplasm (arrow head). **c** Showing severe microvesicular steatosis within the hepatocyte cytoplasm (arrow head). **d** Showing very pale hepatic parenchyma as a result of severe glycogen depletion within the hepatocyte cytoplasm. **e**, **f** Showing severe hemosiderosis distributing within the hepatic parenchyma. **g** Showing severe sinusoidal dilatation with severe congestion (arrow head). **h** Showing severe hemorrhage within the hepatic parenchyma. **i** Showing severe chronic inflammatory reaction representing in chronic inflammatory cell infiltrations with fibrous connective tissue proliferations (arrow head). Stain: **a**, **i** blue Masson’s trichrome, **b**, **h** H&E, **c** Sudan black, **d** PAS, **e**, **f** Perls Prussian blue, **g** mercuric bromophenol blue. Scale bars: **a**, **b**, **c**, **d**, **e**, **f**, **g**, **h** = 40 μm and **i** = 200 μm

On the other hand, the liver of G4 injected intraperitoneal with CuO NPs in a dose of 25 mg/kg bwt for 9 days demonstrated severe and diffuse necrosis of the hepatocytes with completely disorganization of the hepatic cords. The necrotic hepatocytes in G4 were characterized with severe degenerative changes representing in enlarged cell, small pyknotic nuclei, condensed chromatin, lack of nucleolus, very pale and foamy cytoplasm that filled with numerous vacuoles of variable size which were tended to form cystic degeneration (Fig. 3a, b). In addition, severe microvesicular steatosis filling the hepatocyte cytoplasm was appeared (Fig. 3c). With PAS stain, severe glycogen depletions within the hepatocyte cytoplasm were identified giving a very pale hepatic parenchyma (Fig. 3d). With Perls Prussian blue stain, severe hemosiderosis distributing within the hepatic parenchyma was also clarified (Fig. 3e, f).

**Fig. 4:**
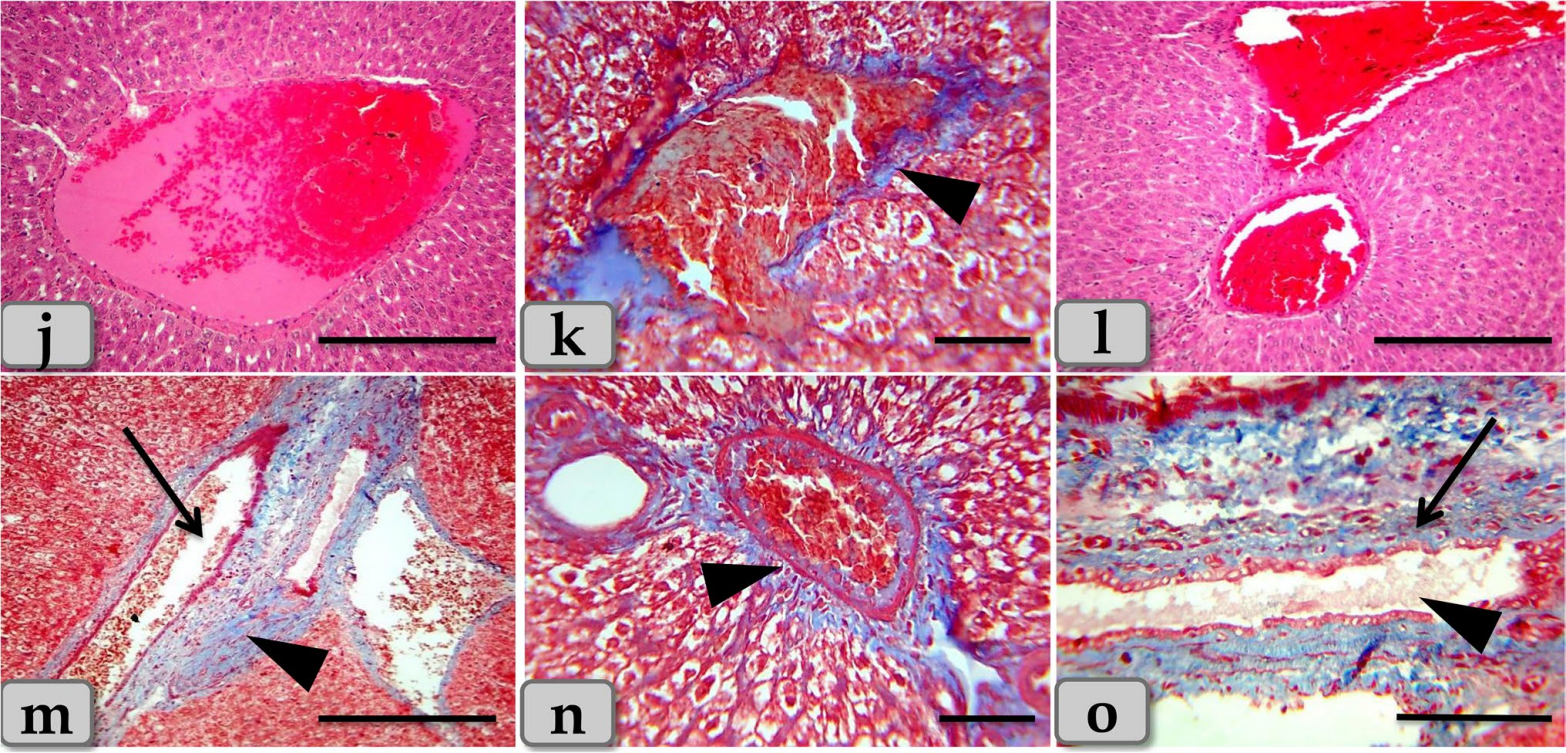
Photomicrographs of the G4, **j** showing severe dilatation and congestion of the central vein. **k** Showing severe necrosis of the lining epithelium of the central vein with severe fibrous connective tissue proliferation; fibrosis surrounding the central vein (arrow head). **l** Showing severe blood vessel dilatation and congestion within the hepatic parenchyma. **m**, **n** Showing severe dilatation and congestion in the blood vessels of the portal area (arrow) with severe chronic inflammatory reaction (arrow head). **o** Showing severe hyperplasia of bile duct (arrow head), surrounding with severe chronic inflammatory reaction (arrow). Stain: **j**, **l** H&E, **k**, **m**, **n**, **o** blue Masson’s trichrome. Scale bars: **j**, **l**, **m** = 300 μm, **o** = 200 μm, and **k**, **n** = 40 μm

And also, severe sinusoidal dilatations and severe congestion (Fig. 3g) with severe interhepatocellular hemorrhage were observed (Fig. 3h). Furthermore, severe chronic inflammatory reactions representing in numerous inflammatory cell infiltrations with fibrous connective tissue proliferations were demonstrated within the hepatic parenchyma (Fig. 3i). Severe dilatation and congestion of the central vein (Fig. 4j), with severe necrosis of its lining epithelium surrounding with severe fibrous connective tissue proliferation; fibrosis were noticed (Fig. 4k). Severe blood vessel dilatation and congestion within the hepatic parenchyma especially in portal area surrounding with severe chronic inflammatory reaction were recognized (Fig. 4l, m, n). Severe enlargement and hyperplasia of bile duct surrounding with severe chronic inflammatory reaction were distinguished (Fig. 4o).

**Fig. 5:**
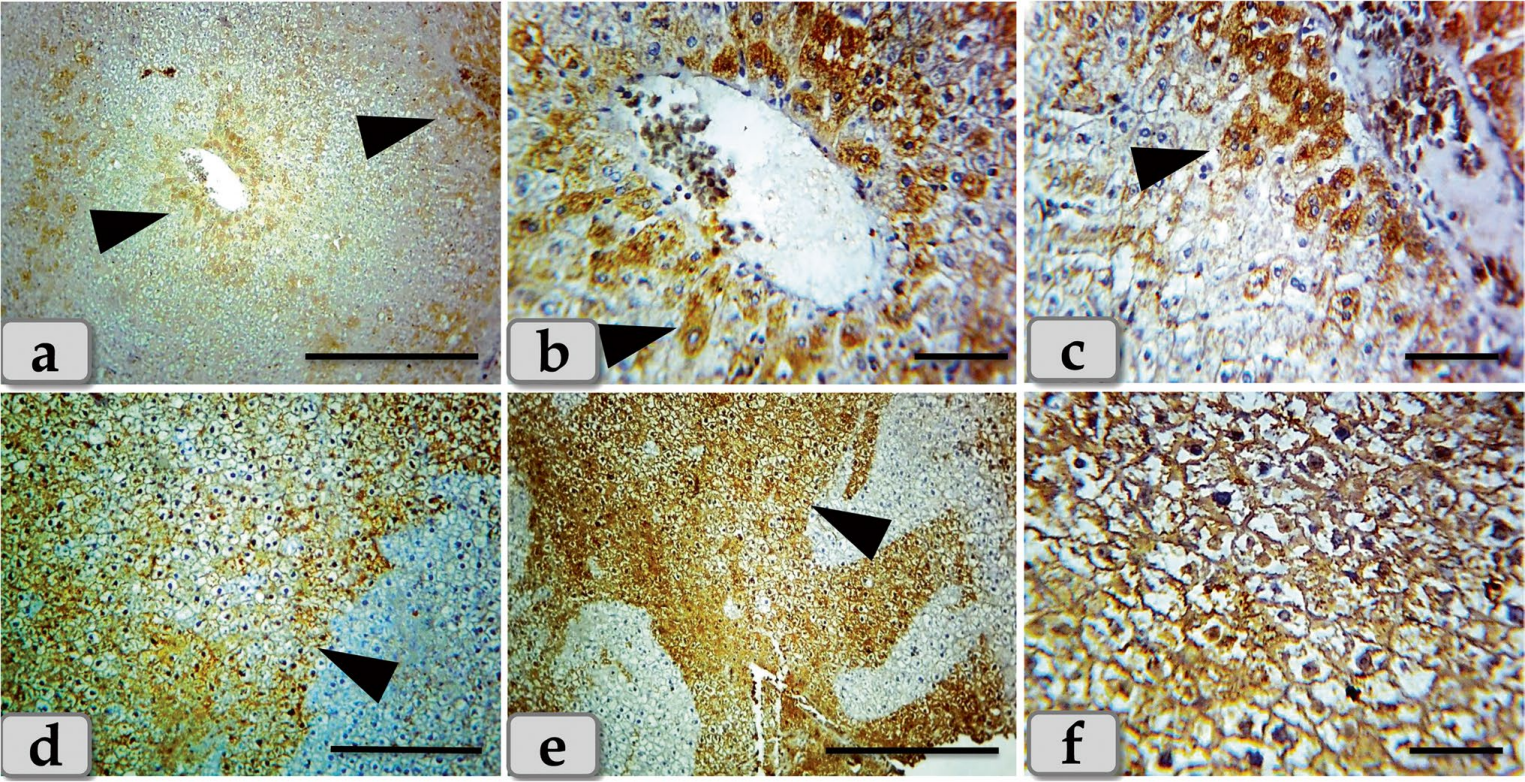
Photomicrographs of the immunohistochemical stain of liver against anti-caspase-3 antibody, **a–c** showing moderate immunoreactivity against anti-caspase-3 antibody (arrow head) of G2 and G3 that was only expressed in the nucleus and cytoplasm of the apoptotic hepatocytes specially around the central vein and portal triad. **d–f** Showing diffuse immuno-localization against anti-caspase-3 antibody (arrow head) of G4 that were widely expressed in almost of the hepatic parenchyma confirming widespread of apoptosis. Stain: **a–f** immunohistochemical stain against anti-caspase-3 antibody. Scale bars: **a**, **e** = 300 μm, **d** = 200 μm, **b**, **c**, **f** = 40 μm

Concerning immunohistochemical reactivity of liver against anti-caspase-3 antibody, G2 and G3 exhibited moderate immunoreactivity of caspase-3 that was only expressed in the nucleus and cytoplasm of the apoptotic hepatocytes specially around the central vein and portal triad (Fig. 5a, b, c). On the other hand, G4 clarified diffuse immuno-localization of caspase-3 that were widely expressed in almost of the hepatic parenchyma confirming widespread of apoptosis (Fig. 5d, e, f).

The histological examination of the mature Westar rats’ kidney of the control group (G1) clarified normal, intact renal parenchyma without any pathological changes. Renal parenchyma was organized into two main portions: the outer cortex and inner medulla. This parenchyma was composed mainly of numerous uriniferous tubules that composed of functional units (nephrons) continuous with a system of collecting tubules.

**Fig. 6:**
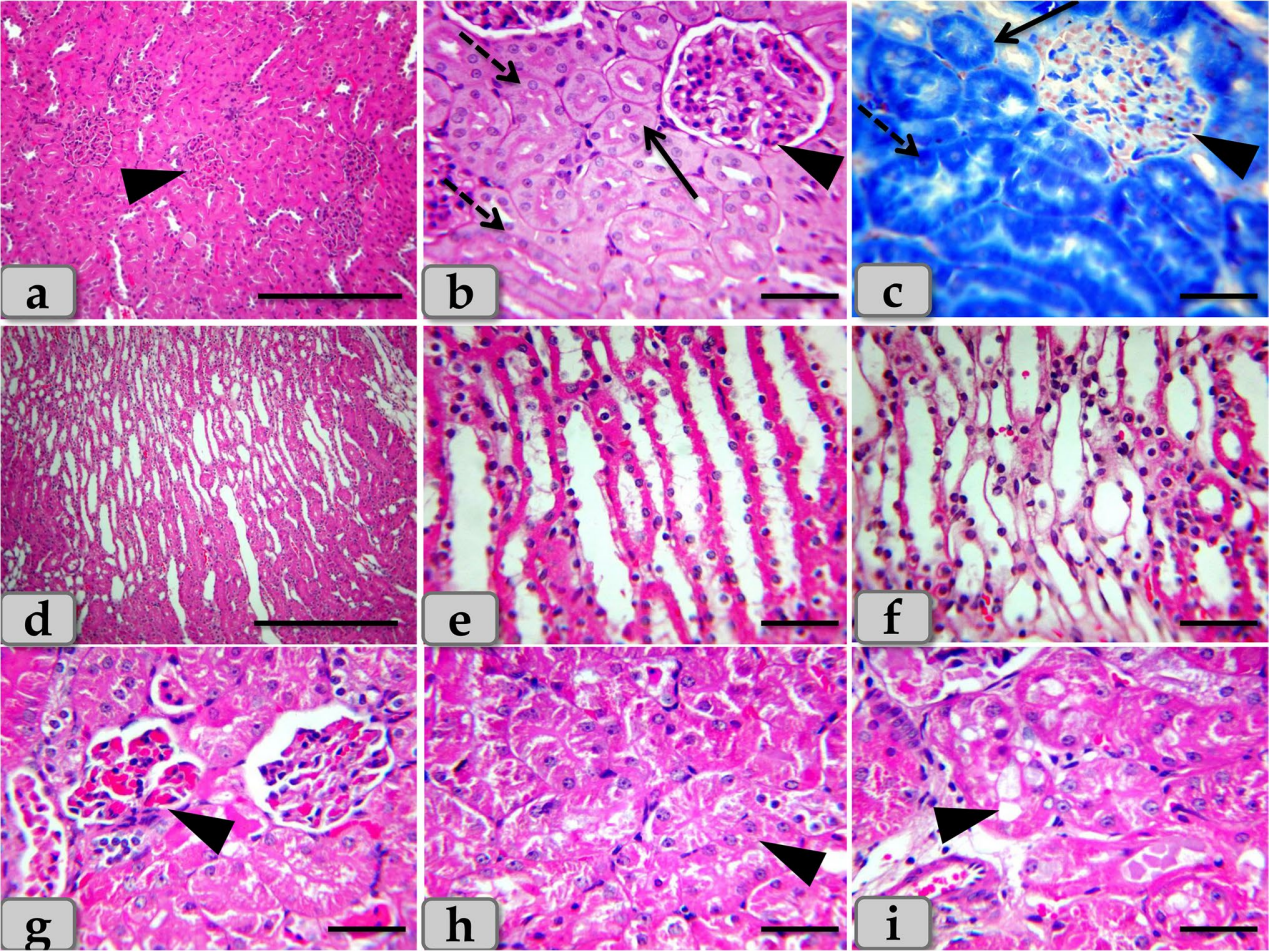
**a–f** Photomicrographs of the mature Westar rats’ kidney of the control group (G1), **a** showing normal, intact renal cortex; renal corpuscles (arrow head), surrounding with normal PCT; proximal convoluted tubules and DCT; distal convoluted tubules. **b**, **c** Showing intact renal corpuscle (arrow head) surrounding with intact PCT (arrow) and DCT (dashed arrow) with intact lining epithelium. **d–f** Showing intact renal medullary rays; medullary tubules with intact lining epithelium. **g–i** Photomicrographs of G2 and G3, **g** showing moderate necrosis and atrophy of the renal corpuscles with damage of Bowman’s capsules and glomerulus with its lining epithelium, in addition, moderate congestion of the glomerulus (arrow head). **h** Showing moderate necrosis of the renal tubules with moderate damage in its lining epithelium (arrow head). **i** Showing vacuolations of different shape and size of renal tubules lining epithelium (arrow head). Stain: **a**, **d**, **e**, **f**, **g**, **h**, **i** H&E, **b** PAS, **c** mercuric bromophenol blue. Scale bars: **a**, **d** = 300 μm and **b**, **c**, **e**, **f**, **g**, **h**, **i** = 40 μm

The cortex was composed of numerous renal corpuscles; a spherical expansions of the nephron proximal end and deeply invaginated to form double-walled cup-shaped structure; Bowman’s capsules. The cup concavity was occupied by a tuft of glomerulus capillaries. Bowman’s capsule was a double-walled cup; the inner layer was lined with podocytes (modified cuboidal epithelium enveloping the glomerulus capillaries). Meanwhile, the outer layer was lined with simple squamous epithelium. Surrounding the renal corpuscle, groups of tube-like structures were collected: proximal convoluted tubules that were lined with 3–5 simple cuboidal cells with single central spherical nucleus and acidophilic granular cytoplasm and distal convoluted tubules that were lined with 5–8 simple cuboidal cells with single spherical nucleus located near the lumen and less acidophilic cytoplasm (Fig. 6a, b, c).

The renal medulla was composed of numerous “medullary rays”; bundles of straight tubules; collecting ducts lined with cuboidal epithelium and loops of Henle lined with simple squamous epithelium in descending limb and simple cuboidal epithelium in ascending limb (Fig. 6d, e, f).

Regarding the histological examination, the kidney of G2 and G3 characterized with moderate necrosis and atrophy of the renal corpuscles accompanied with damage of Bowman’s capsules and glomerulus with its lining epithelium, in addition, moderate congestion of the glomerulus (Fig. 6g). And also, mild to moderate necrosis of the renal tubules with moderate damage in its lining epithelium accompanied with loss of its brush border (Fig. 6h) and vacuoles of different shape and size within the renal tubules lining epithelium were also noticed (Fig. 6i).

**Fig. 7:**
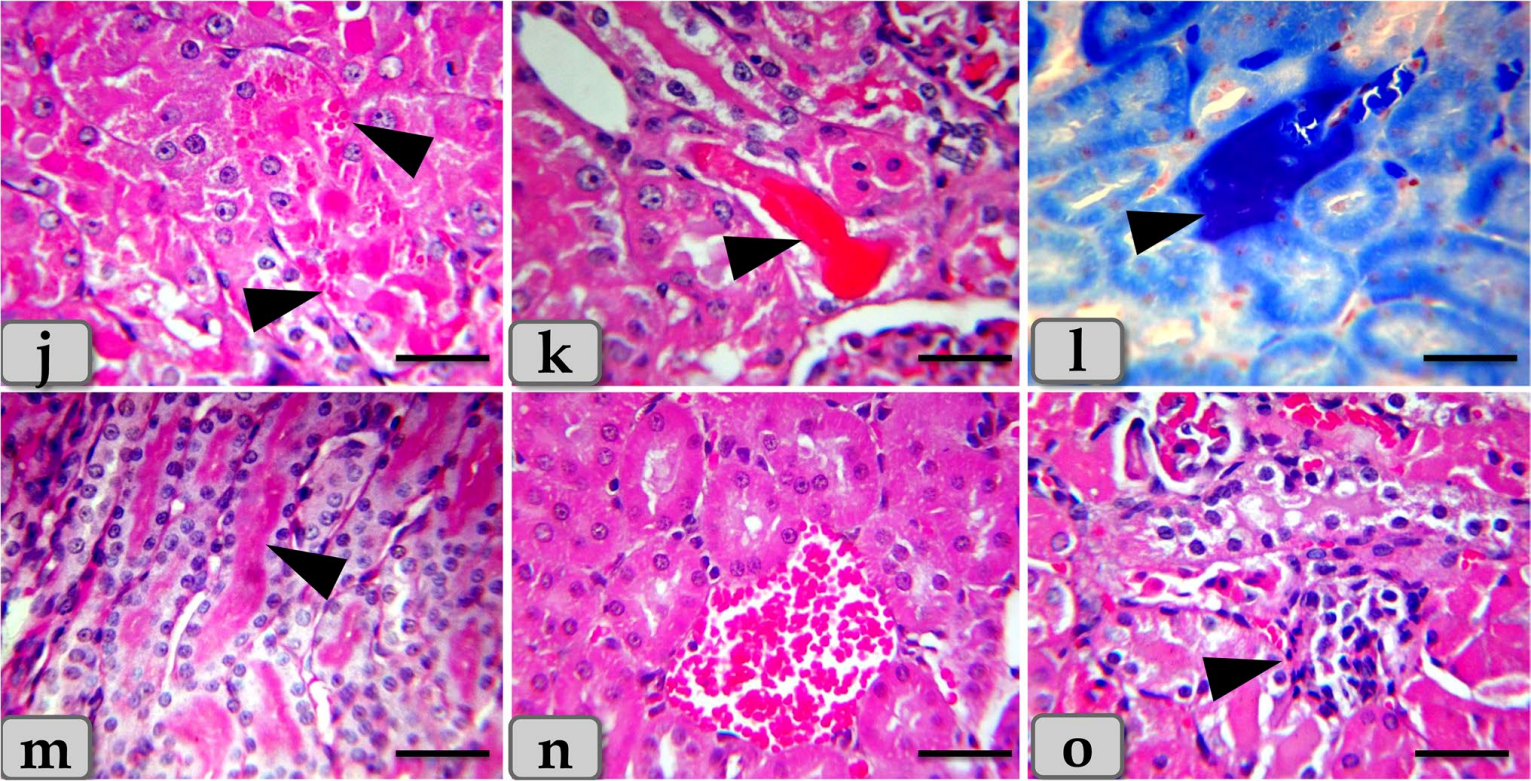
**j–o** Photomicrographs of G2 and G3, **j** showing moderate accumulations of acidophilic granules of variable shape and size in the renal tubules lining epithelium and also in the lumen of renal tubules (arrow head). **k**, **l** Showing accumulations of acidophilic mass in the intertubular areas (arrow head). **m** Showing accumulations of PAS positive material filling the lumen of the renal tubules (arrow head). **n** Showing moderate hemorrhage of the intertubular parts. **o** Showing moderate chronic inflammatory cell infiltrations in between the renal tubules (arrow head). Stain: **j**, **k**, **n**, **o** H&E, **m** PAS, **l** mercuric bromophenol blue. Scale bars: **j–o** = 40 μm

Acidophilic granules of variable shape and size were accumulated within the renal tubules lining epithelium and extended to the lumen of renal tubules (Fig. 7j). In addition, accumulations of acidophilic mass were also observed in the intertubular areas (Fig. 7k, l). The accumulated material was reacted positively with PAS (Fig. 7m). Furthermore, moderate hemorrhage in intertubular parts was distinguished (Fig. 7n). And also, focal scattering of inflammatory cell infiltrations was clarified in between the tubular parts (Fig. 7o).

**Fig. 8:**
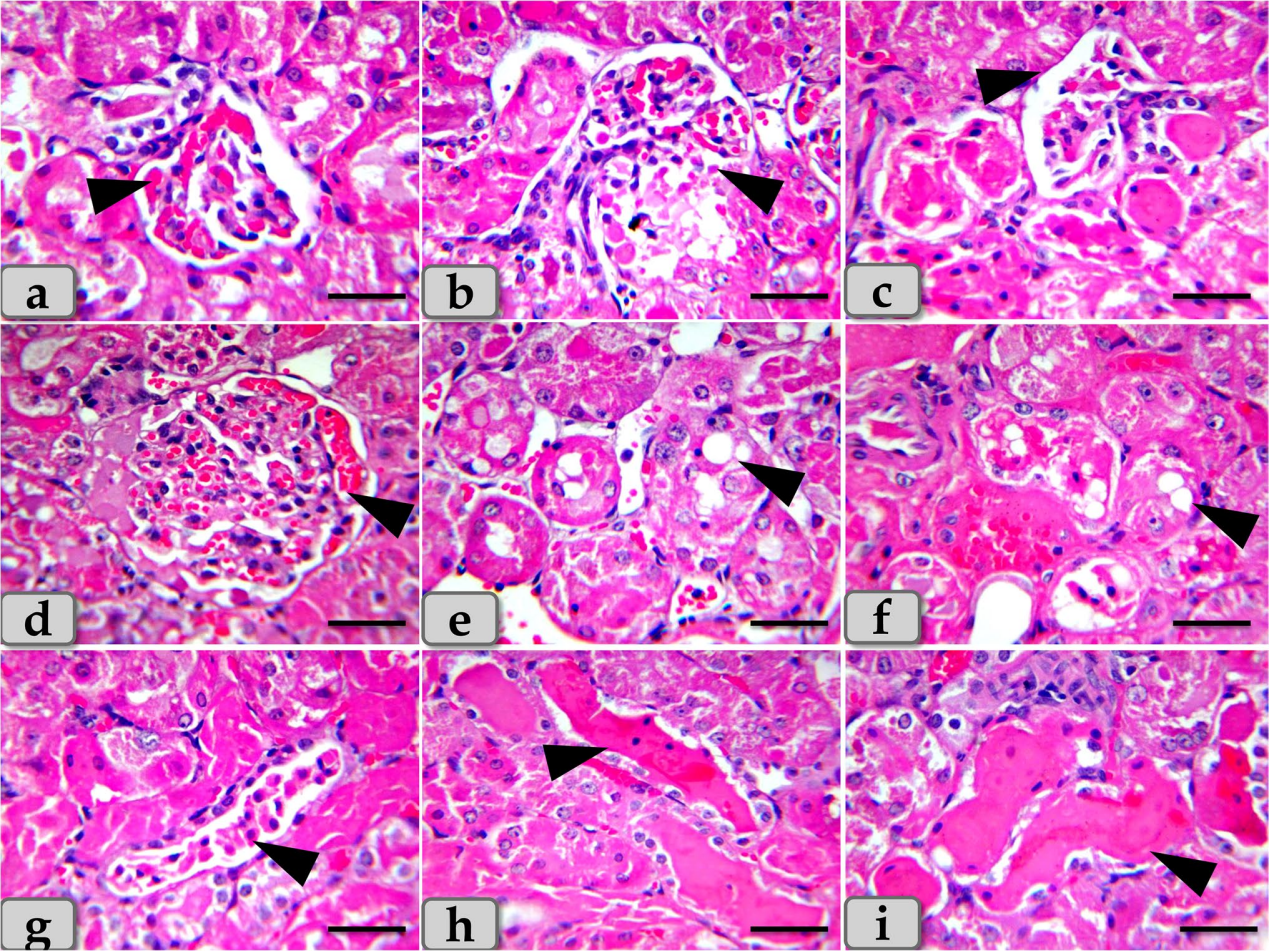
Photomicrographs of the G4. **a–c** Showing severe necrosis and atrophy of the renal corpuscles with damage of Bowman’s capsule lining epithelium with disorganization and loss of normal renal cortex architectures (arrow head). **d** Showing severe congestion of the glomerulus (arrow head). **e**, **f** Showing severe vacuolations of large size distributing inside the renal tubules lining epithelium (arrow head). **g** Showing severe necrosis of the renal tubules with severe damage and sloughing for its lining epithelium with loss of its brush border (arrow head). **h**, **i** Showing severe accumulations of acidophilic material in intratubular parts filling the lumen of renal tubules (arrow head). Stain: **a**–**i** H&E. Scale bars: **a**–**i** = 40 μm

On the other hand, the histological examination of the kidney of the G4 demonstrated severe necrosis and atrophy of the renal corpuscles with severe damage of Bowman’s capsule lining epithelium with completely disorganization and loss of normal renal cortex architectures (Fig. 8a, b, c) with severe congestion of the glomerulus (Fig. 8d).

**Fig. 9:**
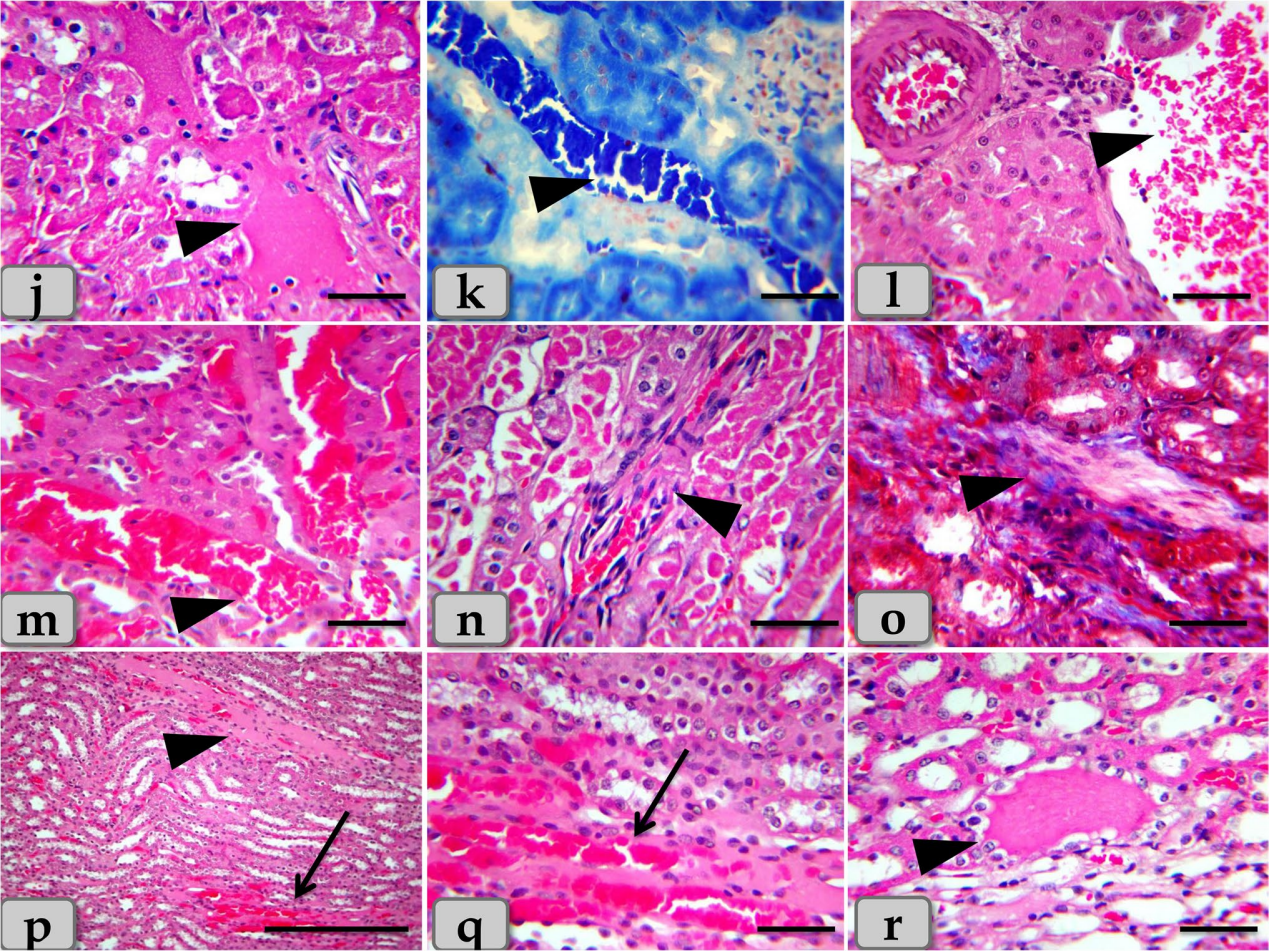
Photomicrographs of the G4. **j** Showing severe accumulations of acidophilic material in intertubular areas filling almost of spaces between renal tubules (arrow head). **k** Showing severe blood vessel dilatation and severe congestion among renal tubules (arrow head). **l**, **m** Showing severe hemorrhage between renal tubules (arrow head). **n** Showing severe chronic inflammatory cell infiltrations between renal tubules (arrow head). **o** Showing severe fibrosis; fibrous connective tissue proliferations between renal tubules (arrow head). **p**–**r** Showing severe congestion and hemorrhage in the medullary rays (arrow) and also accumulation of acidophilic materials within the lumen of medullary tubules with necrosis and sloughing of its lining epithelium (arrow head). Stain: **j**, **l**, **m**, **n**, **p**, **q**, **r** H&E, **o** blue Masson’s trichrome, **k** mercuric bromophenol blue. Scale bars: all = 40 μm except **p** = 300 μm

Moreover, severe vacuolations of large size distributing inside the renal tubules lining epithelium (Fig. 8e, f) with severe necrosis of the renal tubules with severe damage and sloughing for its lining epithelium with loss of its brush border were observed (Fig. 8g). Severe accumulations of large acidophilic masses in intratubular parts filling the lumen of renal tubules (Fig. 8h, i) and also in the intertubular areas filling almost of spaces between renal tubules were demonstrated (Fig. 9j).

Severe blood vessel dilatation and congestion among renal tubules (Fig. 9k) with severe hemorrhage between renal tubules were noticed (Fig. 9l, m).

Severe chronic inflammatory cell infiltrations (Fig. 9n) with severe fibrosis; fibrous connective tissue proliferations between renal tubules were appeared (Fig. 9o). Regarding renal medulla, severe congestion and hemorrhage in the medullary rays and also accumulation of acidophilic materials within the lumen of medullary tubules with necrosis and sloughing of its lining epithelium were clarified (Fig. 9p, q, r).

**Fig. 10:**
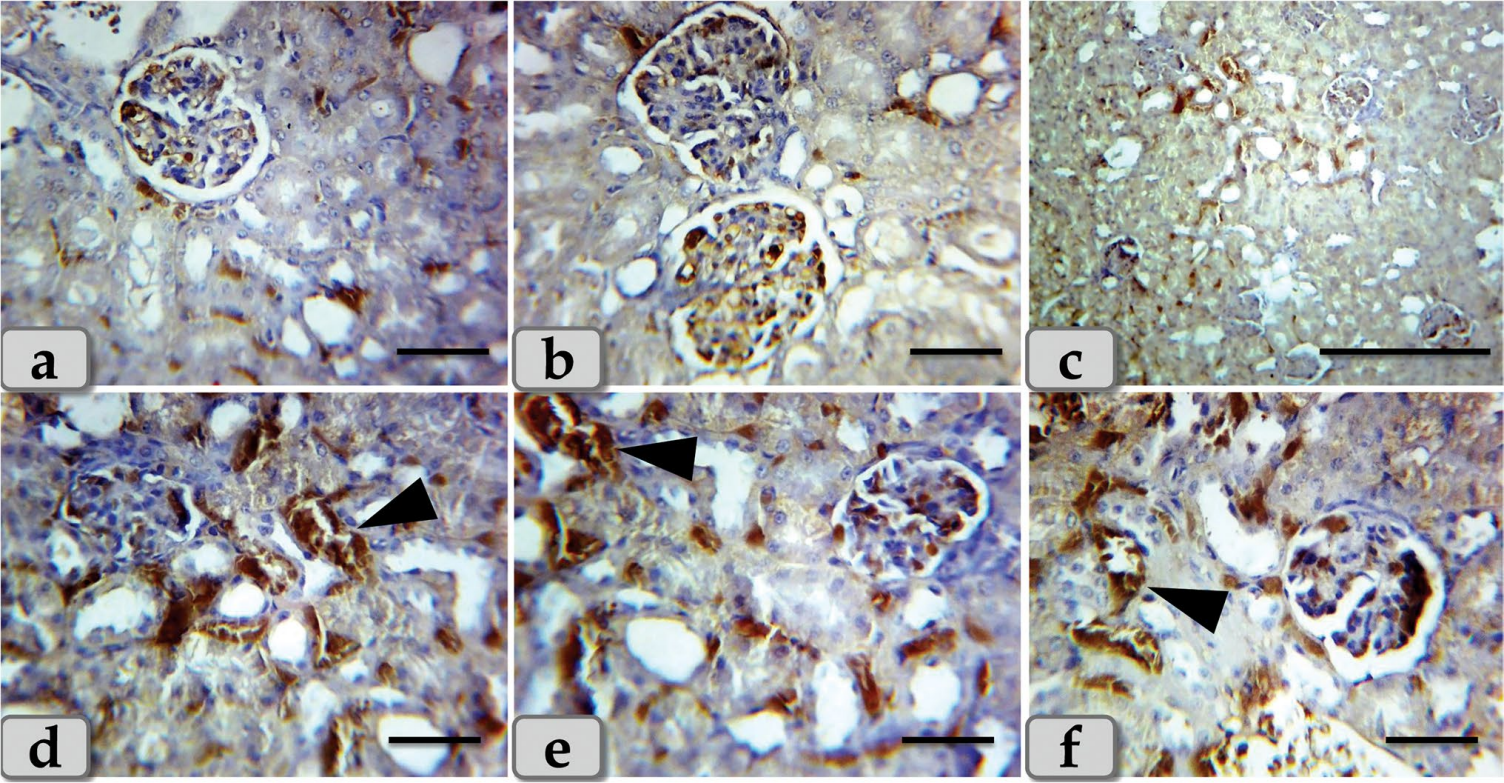
Photomicrographs of the immunohistochemical stain of kidney against anti-caspase-3 antibody, **a**–**c** showing mild to moderate immunoreactivity against anti-caspase-3 antibody of G2 and G3, localized mainly in the renal corpuscles and renal tubules lining epithelium. **d**–**f** Showing strongly immuno-localization against anti-caspase-3 antibody of G4 that is mainly expressed within the renal tubules lining epithelium (arrow head). Stain: **a**–**f** immunohistochemical stain against anti-caspase-3 antibody. Scale bars: **a**, **b**, **d**, **e**, **f** = 40 μm, **c** = 300 μm

Concerning immunohistochemical reactivity of kidney against anti-caspase-3 antibody, G2 and G3 showed mild to moderate immunoreactivity against anti-caspase-3 (stained immunoreactive cells) that were mainly localized in the renal corpuscles and the renal tubules lining epithelium (Fig. 10a, b, c). Meanwhile, G4 clarified severe immuno-localization against anti-caspase-3 that was mainly expressed within the renal tubules lining epithelium (Fig. 10d, e, f).

## Discussions

Nowadays, the utilizations of CuO NPs are elevated in several purposes like biomedicines, gas sensors, industrial catalyst, electronic materials, and environmental remediation because of their flexible characteristics, such as large surface area to volume ratio. These wide usages have raised the human exposure and therefore the hazard potency regarding to their long- and short-term toxicity. The releasing of these NPs to the environment has induced major attentions that become a prominent area of research and development (Naz et al. 2020).

Histologically, the examination of liver of G1 (control group) clarified normal, intact, homogenous hepatic parenchyma without any abnormalities. On the other hand, the histological examination of liver of G2 and G3 injected intraperitoneal with CuO NPs in a dose of 5 mg/kg bwt and 10 mg/kg bwt for 9 days respectively characterized with mild to moderate necrosis of the hepatic parenchyma with disorganization of the hepatic rays. These finding is in agreement with Doudi and Setorki (2014) in rats who clarified the disappearance of hexagonal liver lobules in all treated groups receiving different CuO NP dosages. Furthermore, our result at the level of hepatocytes, the necrosis was distinguished and represented in enlarged cell, pyknotic nucleus, and pale cytoplasm. However, vacuoles with various shape and size were observed distributing within the hepatocyte cytoplasm. The vacuole contents were reacted positively with Sudan black stain confirming fatty changes; microvesicular steatosis; fat droplet aggregations within the hepatocyte cytoplasm. In addition, central vein and sinusoidal dilatations with congestions were also observed. These investigations are in parallelism with Gupta et al. (2016) in common carp (*Cyprinus carpio*) who discovered an increase in the sinusoidal space, hepatocytes with pyknotic nuclei, and the appearance of cytoplasmic vacuoles in the treated groups with lower doses, indicating early stages of necrosis. Furthermore, Chen et al. (2006) in mice described the presence of steatosis around venae centrals of hepatic tissue at a medium dose of CuO NPs. In addition, Yaqub et al. (2018) in albino mice (*Mus musculus*) distinguished that the sub-lethal doses of CuO NPs, in liver, led to sinusoid spaces dilatations, hepatocytes rupture, central vein congestion, and hemorrhaging of the hepatic parenchyma.

Our results revealed moderate hemosiderosis (hemosiderin pigment precipitation) within the hepatocyte cytoplasm with chronic inflammatory reaction distributing in between the hepatocytes and surrounding the portal area with moderate fibrosis in the G2 and G3 receiving CuO NPs in a dose of 5 mg and 10 mg /kg bwt. These findings are completely similar to the findings described after Alferah (2018) in Westar rats.

On the other hand, elevating the dose of CuO NPs, the liver toxicity became more distinct; the liver of G4 injected intraperitoneal with CuO NPs in a dose of 25 mg/kg bwt for 9 days demonstrated severe and diffuse necrosis of the hepatocytes with completely disorganization of the hepatic cords. The necrotic hepatocytes in G4 were characterized with severe degenerative changes representing in enlarged cell, small pyknotic nuclei, very pale and foamy cytoplasm that filled with numerous and variable vacuoles which had a tendency to organize cystic degeneration. In addition, severe microvesicular steatosis (fatty changes), severe hemosiderosis, severe sinusoidal dilatation with congestion accompanied with severe blood vessel congestion within the portal area, severe chronic inflammatory reactions, and fibrosis were also clarified. These investigations were confirmed by the findings of Elhussainy and El-Shourbagy (2014) in rats who mentioned that the group 3 treated with CuO NPs (toxic group) exhibited fatty changes and necrosis in hepatocytes with severe inflammatory cell infiltrations in the portal areas and severe blood vessel congestion within portal tracts. Furthermore, Alferah (2018) in Westar rats and Gupta et al. (2016) in the common carp (*Cyprinus carpio*) demonstrated that the group treated with high dose revealed extensive liver damage as severe necrotic hepatocytes with pyknotic nucleus or cell with dead nucleus, also, damaged blood vessel. Also, Ostaszewska et al. (2016) claimed that fishes exposed to Ag NPs and Cu NPs clarified blood vessel congestions, sinusoidal dilatations, and heterochromatic nuclei of liver.

Furthermore, Griffitt et al. (2007) and Al-Bairuty et al. (2013) in fish and Ibrahim et al. (2015) in rats supported our findings of severe hepatocellular necrosis where they demonstrated that the liver of treated group with high dose showed focal area of hepatocellular necrosis infiltrated by mononuclear cells and polyploidy hepatocytes represented by hepatic cytokaryomegaly accompanied with Kupffer cell activations as well as apoptosis. Hyperplasia of biliary epithelium, formation of newly formed bile ductules in addition to periportal sporadic hepatic cell necrosis and apoptosis were clarified. In addition, El Bialy et al. (2020) in mice supported our investigations where animals received biologically synthesized CuO NPs by using *Ulva fasciata* (macro green alga) that was collected from shallow water showed various pathological alterations in hepatic tissue such as lymphocytic infiltration around necrotized cells, Kupffer cell activation, congestion, and hepatocyte pathological changes. The changes ranged from hydropic degeneration and vacuolization to cell necrosis, loss of cellular boundaries, more eosinophilic cytoplasm, karyorrhexis, and some cells lost their nucleus. Hepatic tissues of mice administered with chemically synthesized CuO NPs showed hydropic degeneration, fatty changes, and Kupffer cell activation. Moreover, Tohamy et al. (2022) elucidated that rats exposed to CuO NPs showed hepatic and renal toxicities expressed in deterioration of serum biochemical, oxidative stress, inflammatory, histopathological, and immunohistochemical markers.

Concerning immunohistochemical reactivity of liver against anti-caspase-3 antibody, G2 and G3 exhibited moderate immunoreactivity of caspase-3 that was only expressed in the nucleus and cytoplasm of the apoptotic hepatocytes specially around the central vein and portal triad. On the other hand, G4 clarified diffuse immuno-localization of caspase-3 that was widely expressed in almost of the hepatic parenchyma confirming widespread of apoptosis. These findings were confirmed by El Bialy et al. (2020) who clarified that the hepatocytes of mice administered biologically synthesized CuO NPs showed marked caspase-3 immunostaining especially around the central vein. And also, hepatocytes of mice administered chemically synthesized CuO NPs showed a moderate degree of caspase-3 immunostained.

Regarding the histological examination, the kidney of G2 and G3 characterized with moderate necrosis and atrophy of renal corpuscles, damage of Bowman’s capsules, swelling and congestions of the glomerulus, mild to moderate necrosis of the renal tubules with moderate damage in its lining epithelium accompanied with loss of its brush border. And also, acidophilic granules of variable shape and size were accumulated within the renal tubules lining epithelium and extended to the lumen of renal tubules. Our results were supported by Privalova et al. (2014) who demonstrated that obvious necrotic and degenerative change of epithelial cells lining the renal tubules, partial damage of the brush border, small granules of a pigment in the renal tubules lining epithelial cells, and tubular lumen were noticed. In addition, Yaqub et al. (2018) in the albino mice (*Mus musculus*) distinguished that the sub-lethal doses of CuO NPs revealed loss of urinary space, damage of the renal capsule, podocyst degeneration, glomerulus swelling, and cytoplasmic vacuolizations.

On the other hand, with increasing the dose of CuO NPs, the kidney revealed severe necrosis and atrophy of the renal corpuscles with severe damage of Bowman’s capsule lining epithelium with completely disorganization and loss of normal renal cortex architectures, severe congestion of the glomerulus, severe vacuolations in the renal tubules lining epithelium, severe necrosis of the renal tubules with severe damage and sloughing for its lining epithelium, with severe hemorrhage between renal tubules were noticed. These findings was supported by Elkhateeb et al. (2020) in rats who demonstrated renal tubule swelling, glomerular sclerosis, hyperplasia of the tubular epithelial accompanied with karyomegaly and binucleation, congestion of interstitial blood vessels, and glomeruli sclerosis. Moreover, Elhussainy and El-Shourbagy (2014) in rats mentioned that the group 3 treated with CuO nanoparticles (toxic group) revealed moderate atrophy of some glomeruli and vacuolization (hydropic degeneration of some renal tubules and marked blood vessel congestion). And also, El Bialy et al. (2020) clarified that the renal tissues of mice administered biologically synthesized CuO NPs demonstrated severe coagulative necrosis, glomerular hypercellularity, detached tubular epithelia, loss of brush border, and intraluminal hyaline casts in cortex and medulla. In addition, renal tissues of mice administered chemically synthesized CuO NPs showed glomerular hypercellularity, cloudy swelling, narrowing of tubular lumen, intertubular hemorrhage, pyknotic nuclei, and tubular cell necrosis.

Immunohistochemical reactivity of kidney against caspase-3 antibody revealed moderate immunoreactivity that mainly localized in the renal corpuscles and the renal tubules lining epithelium of G2 and G3. Meanwhile, G4 clarified severe immuno-localization that was mainly expressed within the renal tubules lining epithelium confirming apoptosis. These findings were supported by Elkhateeb et al. (2020) in rats who revealed apoptosis by the immunohistochemical stained with caspase3 reaction.

## Conclusion

The present investigation can conclude that the CuO NPs have a potential toxicological effect on the hepatic and renal tissues and with increasing the dose, they induced severe histopathological damages that may affect their functions.

## Limitations of the study

To investigate the detailed mechanism of CuO NP toxicity, intensive oxidative stress, immunotoxicity, cytotoxicity, and genotoxicity studies should be done in vitro and in vivo in different organisms, and factors influencing the toxicity of CuO NPs including their shape, size, surface modification, morphology, and concentration should also be considered. In future, we suggest that much work should be done in the field of genotoxicity induced by CuO NPs as this area is less explored. Research must be conducted in the context of present risk assessments linked with CuO NPs, their applications, distribution, and release into the environment.

## Data Availability

All data generated during the current study are included in this manuscript.

## Abbreviations

NPs: Nanoparticles
CuO NPs: Copper oxide nanoparticles
